# A Curious Presentation of May-Thurner Syndrome With Isolated Iliofemoral Deep Vein Thrombosis

**DOI:** 10.7759/cureus.22124

**Published:** 2022-02-11

**Authors:** Bereket Tewoldemedhin, Nardos K Tewoldemedhin, Shahzad Ahmed, Sabin Karki, Miriam Micheal

**Affiliations:** 1 Internal Medicine, Lower Bucks Hospital, Bristol, USA; 2 General Practice, Hayat Medical College, Addis Ababa, ETH; 3 Cardiology, Lower Bucks Hospital, Bristol, USA; 4 Internal Medicine, Howard University College of Medicine, Washington DC, USA; 5 Internal Medicine, University of Maryland School of Medicine, Baltimore, USA

**Keywords:** intravascular ultrasound (ivus), proximal massive deep vein thrombosis, systemic anticoagulation, mechanical thrombectomy (mt), may-thurner's syndrome

## Abstract

May-Thurner syndrome, which has been called by many names, including Cockett syndrome, iliocaval compression syndrome, and iliac vein compression syndrome, is an anatomic variation where there is extrinsic venous compression by the arterial system against the bony structure of the axial skeleton, most commonly right common iliac artery compressing the left iliac vein against the fifth lumbar vertebra. The persistent right common iliac pulsation results in endothelial irritation of the venous system and at the same time reduces venous return, hence satisfying two factors in Virchow's triad for the formation of venous thrombosis. Here we present a rare case of a patient who presented with multiple risk factors that could propagate the formation of deep vein thrombosis with the underlying anatomic variation of May-Thurner syndrome in the setting of dehydration, systemic infection, failure to thrive, and psychiatric decompensation. Treatment required fluid hydration, antibiotic therapy, and mechanical thrombectomy in conjunction with local infusion of thrombolytics. Subsequent stent placement was performed to prevent re-thrombosis and stenosis of the affected area with long-term oral anticoagulation.

## Introduction

The exact incidence and prevalence of May-Thurner syndrome continues to be controversial and is stated to be underestimated [1,2]. The reason for this is majority of individuals who have this variation tend to be asymptomatic throughout their lives. It is said to be seen in 2%-5% of patients who present with lower extremity deep vein thrombosis [2]. Risk factors that could increase the formation of clots and progression to the symptomatic disease include postpartum state, oral contraceptive use, dehydration, scoliosis, repeated radiation exposure, and hypercoagulable state [2,3].

## Case presentation

A 57-year-old Caucasian female with a past medical history of hypertension and bipolar disorder with psychotic features was initially brought into the Emergency Department by her partner after she had shown signs of self-neglect and thoughts of self-harm. The patient was disheveled and slow to respond and had been exhibiting the symptoms for more than two weeks. Her significant other stated that she had been "depressed" and had not been getting out of bed for most of the two weeks prior to the presentation. The patient did endorse dull throbbing intermittent pain in the left lower leg. Had also noted left lower leg swelling. The patient did not have any recent travel history or any recent surgeries. At the time, the patient also endorsed urinary urgency, frequency, and dysuria with periumbilical discomfort. Prior medical history was significant for bipolar disorder with psychotic features and had multiple admissions to the behavioral health unit for intents of self-harm. Her home medications included metoprolol tartrate for high blood pressure, lithium carbonate for bipolar disorder and zolpidem for insomnia. Her family history was noncontributory. She had been smoking one pack of cigarettes daily since the age of 16 and denied recreational drug use or alcohol use.

Her vital signs were as follows: temperature, 37.1 °C; heart rate, 95 beats/minute; blood pressure, 113/47 mmHg; respiratory rate, 16 breaths/minute; oxygen saturation, 100% on room air. Physical examination revealed a soft, non-distended abdomen with normal bowel sounds and mild tenderness on the suprapubic region. Left lower extremity was warm, swollen, mildly erythematous from feet up to mid-thigh. Dorsalis pedis and posterior tibialis pulses were normal. Reflexes, strength and sensation were normal. A complete blood count showed a white blood cell count of 18.6 K/µL, hemoglobin 15.8 g/dL, mean corpuscular volume of 93.4 fL and platelet count of 239 K/µL. Automated differential showed a neutrophil predominance of 85% with bands of 9%. The complete metabolic panel showed blood glucose at 108 mg/dL, blood urea nitrogen 126 mg/dL and serum creatinine at 3.3 mg/dL; anion gap was 14.8 mmol/L with hyperosmolar hyponatremia with serum osmolality of 303 mOsm/kg, a serum sodium level of 123 mmol/L and serum chloride level of 86 mmol/L indicating severe volume depletion with acute kidney injury. Drug toxicology screening was negative. Serum lithium levels were within the therapeutic range. Urinalysis showed gross pyuria with leukocyte esterase positivity. Blood cultures and urine cultures grew pan-susceptible *Escherichia coli*. Prothrombin time was 11.5 seconds, activated partial thromboplastin time was 28.0 seconds and international normalization ratio was 1.05 (Table 1).

**Table 1:** Laboratory test results of the patient

	Patient results	Reference values
Hemoglobin	15.8 g/dL	12-16 g/dL
Mean corpuscular volume	93.4 fL	80-98 fL
Platelets	239,000/µL	150,000-400,000/µL
White blood cell	18,600/µL	3100-10,500/µL
Neutrophils	15,800/µL	1500-7000/µL
Neutrophils (relative percent)	85%	40%-60%
Bands	1700/µL	0-900/µL
Bands (relative percent)	9%	0-5%
Lymphocytes	600/µL	1000-4000/µL
Lymphocytes (relative percent)	3%	20%-40%
Monocytes	600/µL	300-900/µL
Monocytes (relative percent)	3%	4%-8%
Glucose	108 mg/dL	70-140 mg/dL
Blood urea nitrogen	126 mg/dL	6.0-24 mg/dL
Creatinine	3.3 mg/dL	0.5-1.0 mg/dL
Sodium	123 mmol/L	136-145 mmol/L
Potassium	4.2 mmol/L	3.5-5.3 mmol/L
Chloride	86 mmol/L	98-110 mmol/L
Bicarbonate	22.2 mmol/L	20-31 mmol/L
Anion gap	14.8 mmol/L	6-19 mmol/L
Serum osmolality	303 mOsm/kg	278-305 mOsm/kg
Activated partial thromboplastin time	28.0 seconds	24.7-31 seconds
Prothrombin time	11.5 seconds	9.7-12.2 seconds
International normalization ratio	1.05	<4

**Figure 1:**
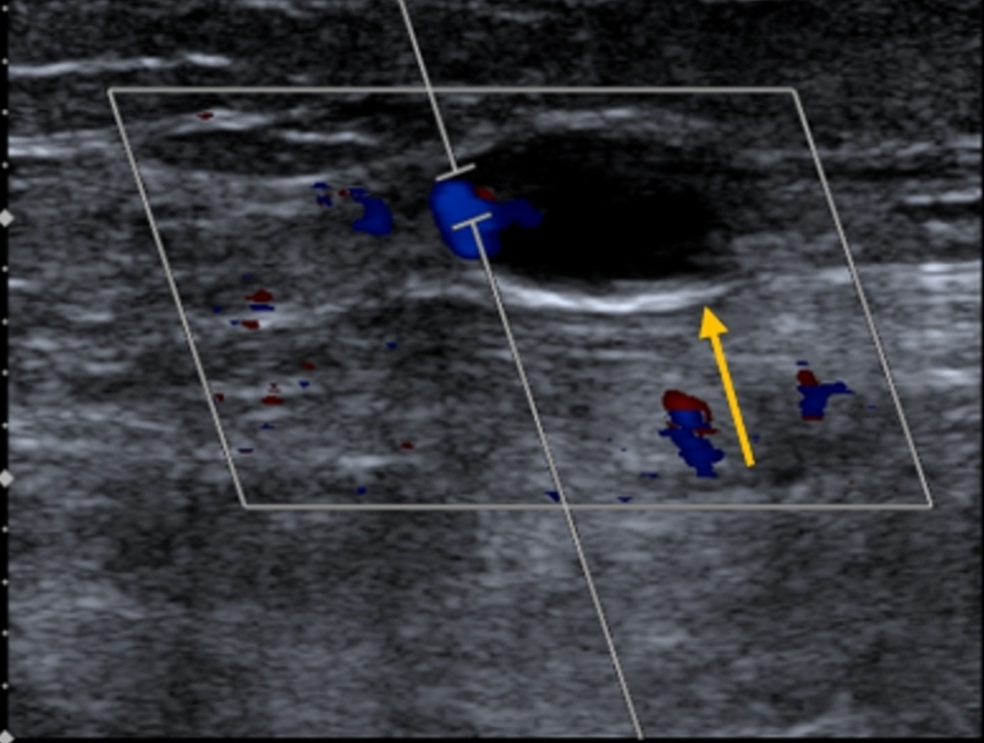
Color Doppler ultrasound demonstrating the near absence of blood flow in the popliteal vein (yellow arrow)

Her complete abdominopelvic ultrasound was unremarkable. Left lower extremity ultrasound revealed extensive near occlusive thrombus extending from the popliteal vein up the common femoral vein evidenced by a near absence of blood flow on the color Doppler study (Figure 1).

**Figure 2:**
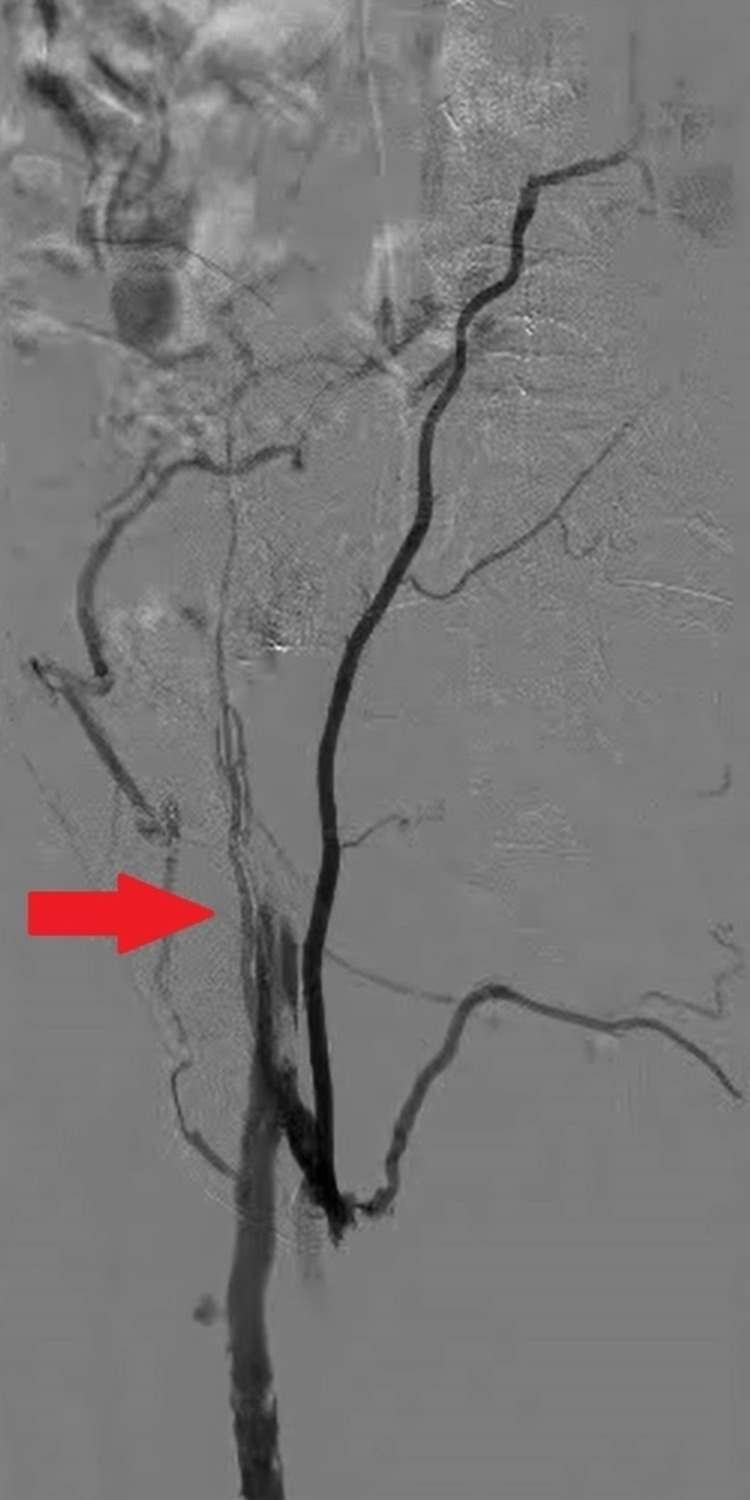
A venogram of the left lower extremity with abrupt cessation of blood flow (red arrow) beyond the common iliac vein with extensive collateral formation

She was aggressively hydrated, and antibiotic coverage was achieved with cefepime. She was placed on full-dose anticoagulation initially with low-molecular-weight heparin, which was subsequently transitioned to unfractionated heparin drip for tight control during the periprocedural time. The left lower extremity venogram revealed 100% occlusion of the left common iliac and external iliac veins (Figure 2).

**Figure 3:**
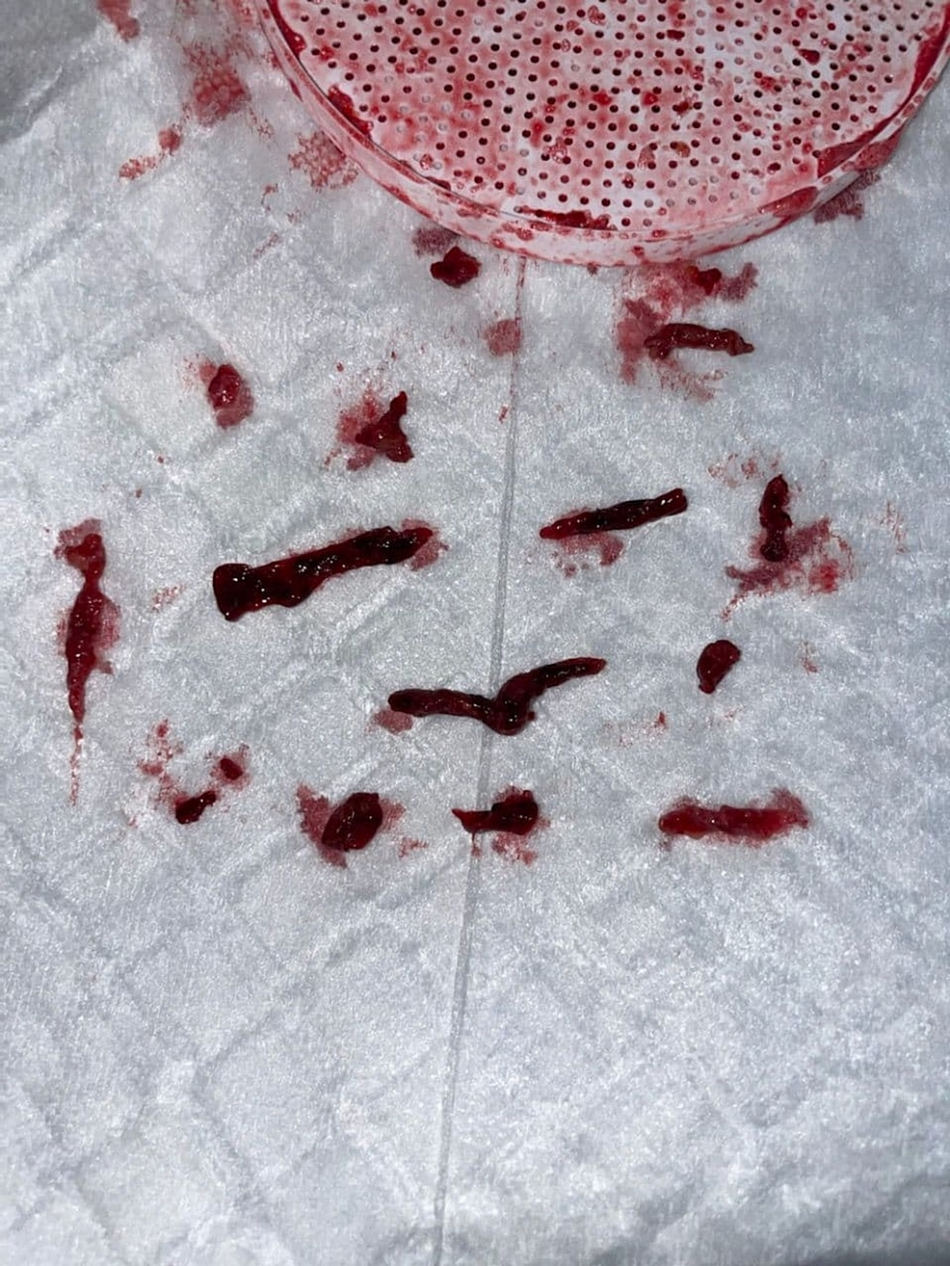
Clot burden removed from the left common iliac vein

A 14-Fr sheath large-bore aspiration catheter was used to remove a huge clot burden (Figure 3).

**Figure 4:**
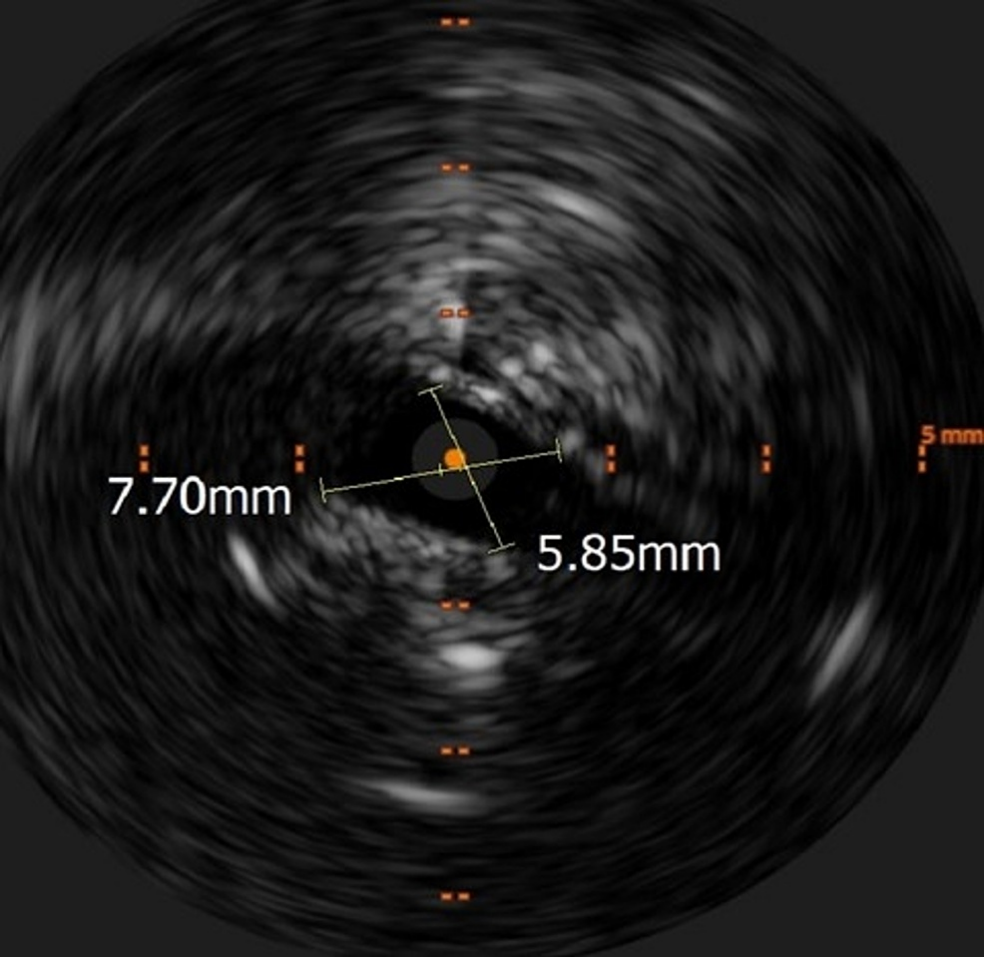
Intravascular ultrasound showing critical luminal narrowing

Intravascular ultrasound of the left iliac and femoral vein performed after clot removal showed critical narrowing of the left common iliac vein; a large amount of residual thrombus was identified within the iliac and femoral veins (Figure 4). An 8-Fr guide catheter was inserted in the left femoral vein through which an 18-h infusion of 1 mg/h tissue plasminogen activator was performed.

**Figure 5:**
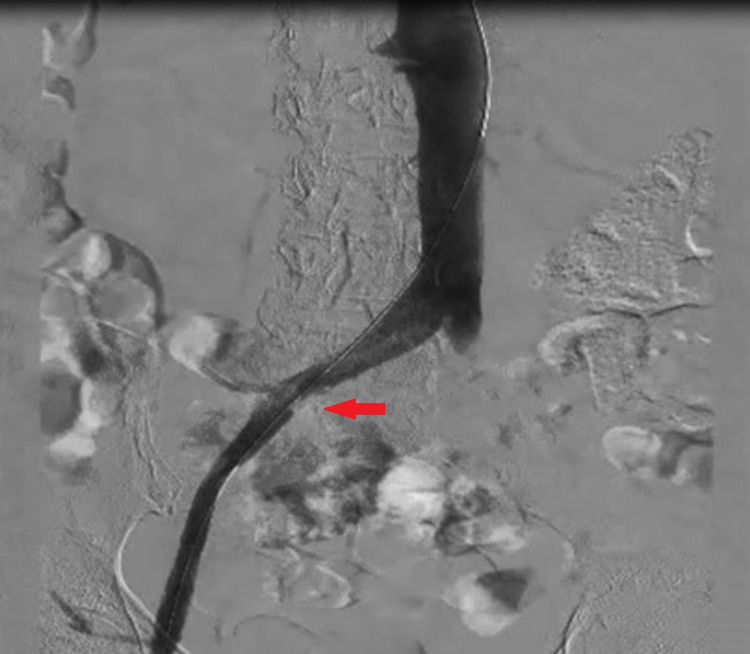
A repeat venogram indicating minimal re-thrombosis distal to the common iliac vein (red arrow)

Following the tissue plasminogen activator infusion, the patient was started on a heparin drip that was titrated to an activated prothrombin time of 50-70 seconds. Repeat venography one day later revealed residual thrombus and stenosis (Figure 5).

**Figure 6:**
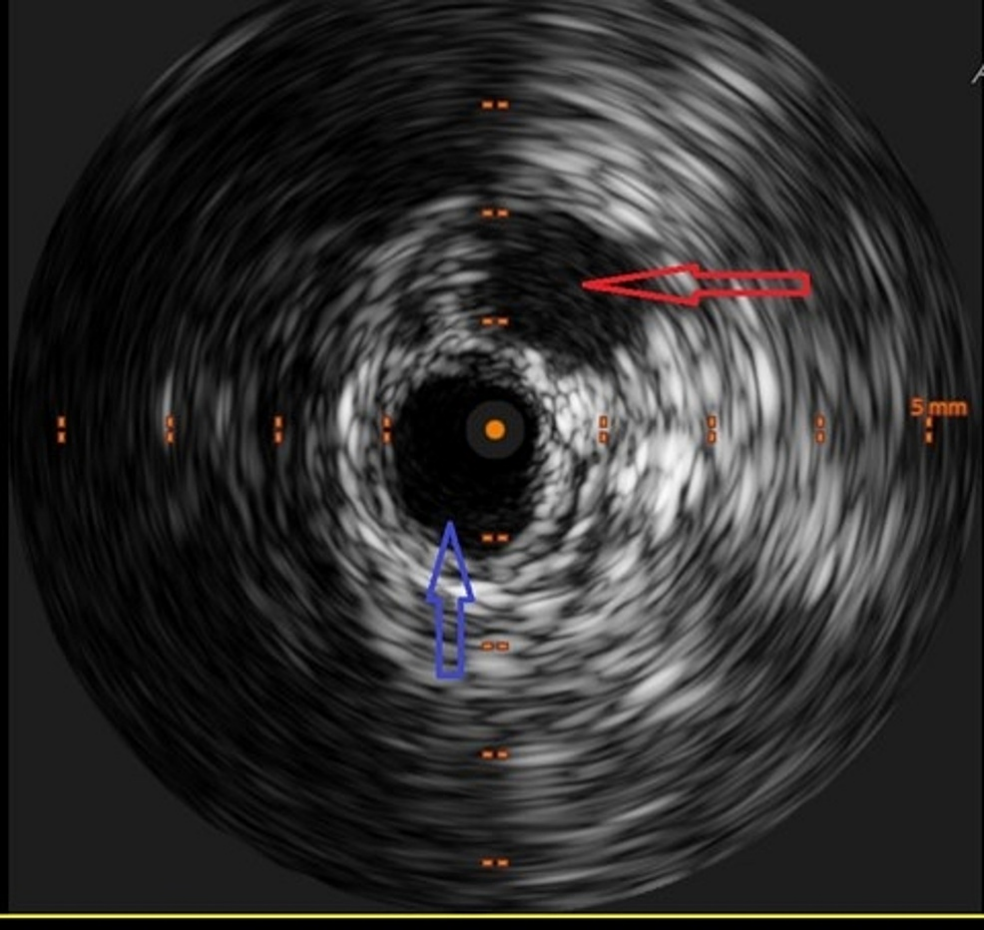
Intravascular ultrasound demonstrating the right common iliac artery (red arrow) abutting the left common iliac vein (blue arrow) typical of the May-Thurner syndrome

Mechanical thrombectomy was performed using an endovascular thrombolysis catheter with an infusion basket. Following the complete removal of thrombus, intravascular ultrasound performed indicated stenosis within the proximal common iliac vein that was dilated by a 14 x 40 mm high-pressure balloon. Intravascular ultrasound also confirmed compression of the left iliac vein with the femoral artery compatible with the May-Thurner physiology (Figure 6).

**Figure 7:**
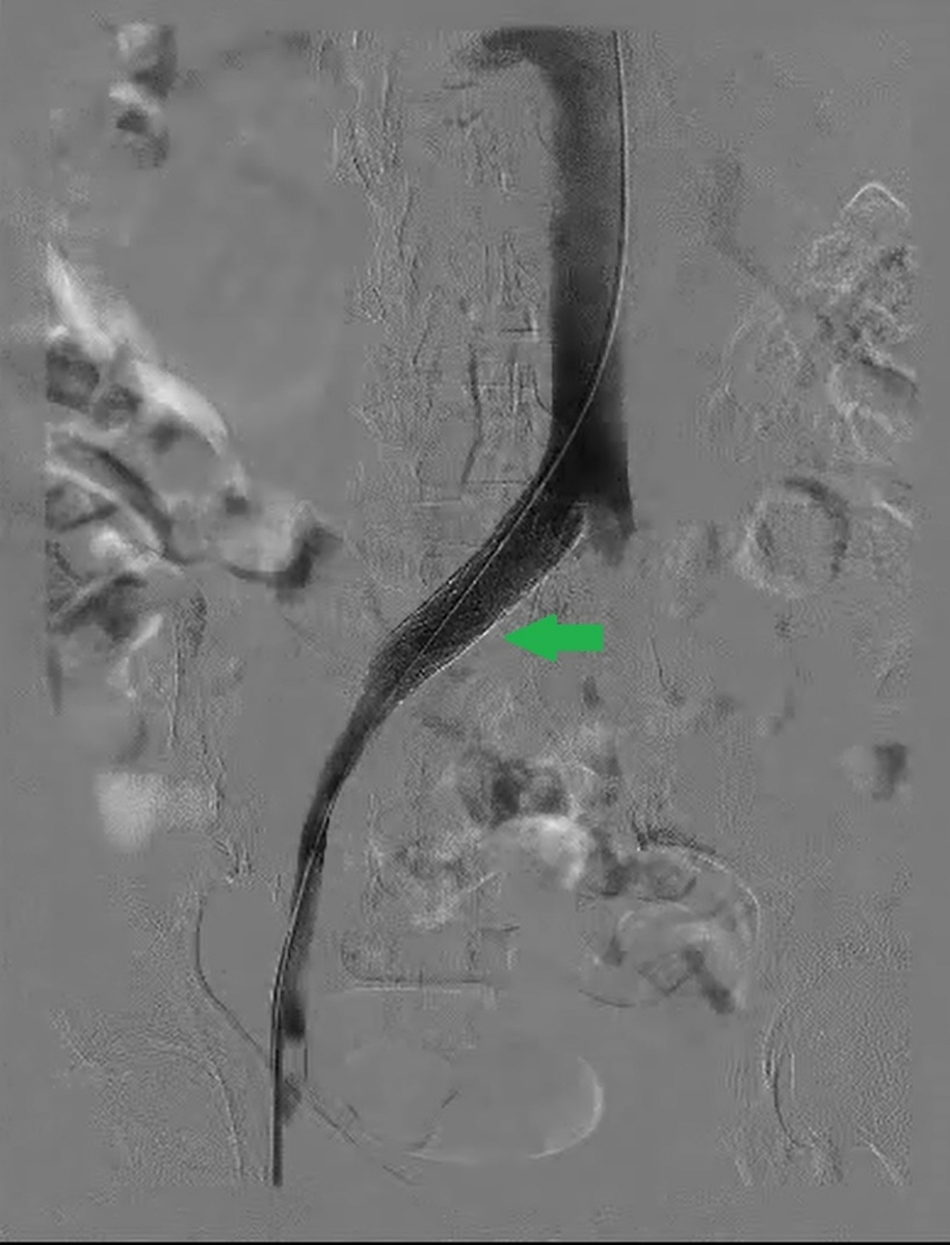
A venogram following stent placement indicating good blood flow and stent position (green arrow)

To prevent recurrent venous thrombosis and keep the stenosis open, a 14 x 60 mm self-expanding venous stent was placed in the proximal left common iliac vein. A venogram was performed to confirm stent placement with no complications (Figure 7).

**Figure 8:**
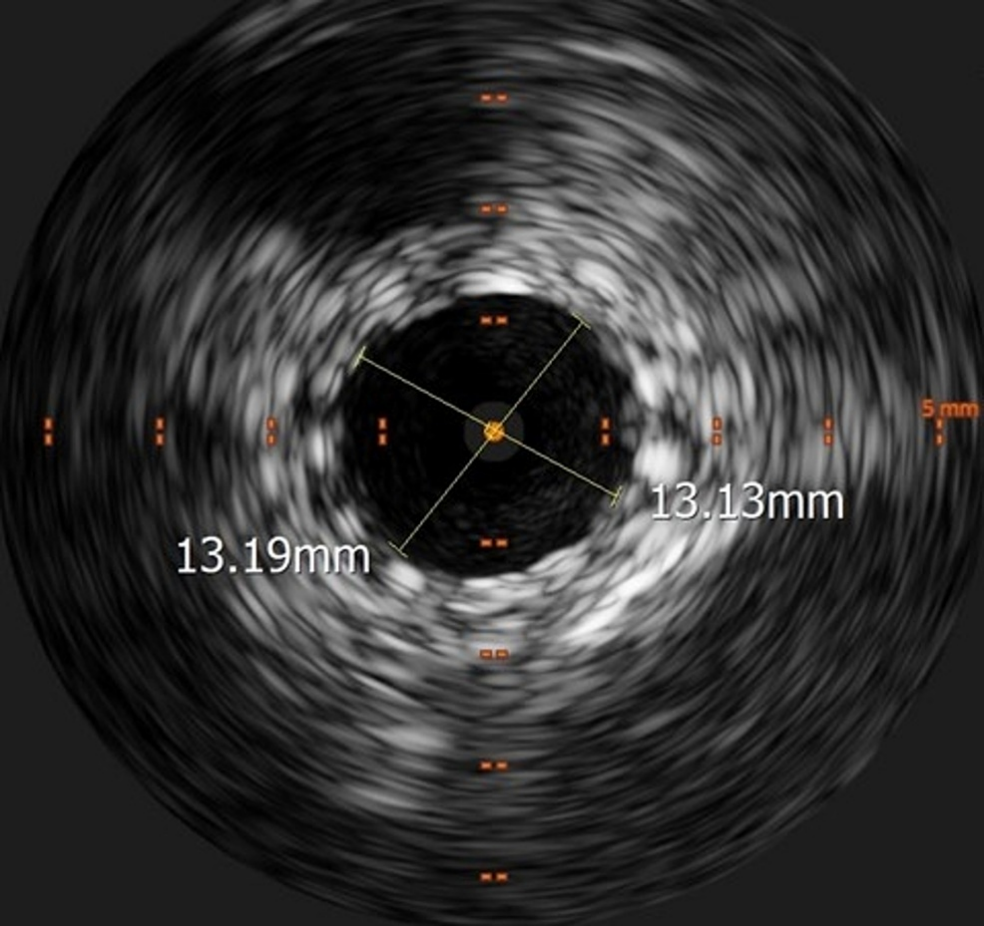
Intravascular ultrasound showing the widely patent left common iliac vein with the surrounding stent

The repeat intravascular ultrasound performed showed no residual stenosis with a well-apposed stent (Figure 8).

The patient tolerated the procedure well with no complications. Following the placement of the stent, she was initiated on aspirin 81 mg daily, with anticoagulant rivaroxaban initiated at 15 mg twice a day for 21 days followed by 20 mg daily for a minimum of 6 months. She was counseled to continue anticoagulation as well as antiplatelet therapy until the completion of hypercoagulability workup that would be done as an outpatient in the Hematology Clinic. She remained afebrile, and repeat blood cultures were clear and were negative for growth; leukocytosis resolved with the white blood cell count down to 12.6 K/µL. Antibiotics were deescalated to oral cephalexin for the completion of treatment. Sodium levels were corrected to 138 mmol/L; acute kidney injury resolved with blood urea nitrogen at 20 mg/dL and creatinine down to 1.0 mg/dL. The patient was subsequently transferred to the behavioral health unit and with an improvement of affect and mentation was discharged in a stable condition.

## Discussion

May-Thurner syndrome is named after May and Thurner who described the anatomy in 1957, and later clinically elaborated by Cockett and Thomas in 1965 [2-5]. It was not a fully understood entity until the mid-20th century when postmortem autopsy of cadavers showed that 22% cases had an anatomic variant where there was an overriding of the right common iliac artery compressing the left common iliac vein against the lumbar spine [5]. This anatomic variation results in chronic irritation of the trapped vein with resultant endothelial irritation and intimal thickening. This coupled with the mechanical venostasis due to the overriding artery predisposes the patients to have deep vein thrombosis and develop post-thrombotic syndrome [6]. As expected, the majority of cases would have classic left-sided deep vein thrombosis. Most tend to be females particularly in the postpartum. The female-to-male ratio is described as high as 5:1 [1,2]. Other risk factors that contribute to the propagation of clots include the use of oral contraceptives, prolonged immobilization, prolonged travel, postsurgical state, severe dehydration with hyper-viscosity, and scoliosis that increases the compression [7]. There have been multiple reports and studies that have used CT scans, and postmortem cadaveric studies trying to delineate the incidence of the pathological variant [8,9]. Overall, it is still estimated that 2%-3% of lower extremity deep vein thrombosis is attributed to May-Thurner syndrome [10].

Clinical presentation of May-Thurner syndrome is variable depending on the stage of the disease and has been described to develop through three stages [10]. Stage I is asymptomatic left common iliac vein compression. Stage II is considered with the presence of intraluminal thickening that projects within the lumen of the vein, the so-called venous spurs that appear to be a direct result of the external compression of the vein. And finally, Stage III would be a left iliac deep vein thrombosis. Depending on the stage and extent of thrombus, patients may present with unilateral leg swelling, pain, venous claudication, or varicosities [10,11]. Some may also present with phlebitis, post-thrombotic syndrome, and the rare limb-threatening complication termed phlegmasia cerulea dolens, seen in the setting of massive iliofemoral deep vein thrombosis with near-total or total thrombotic occlusion of venous drainage and microvascular collaterals [11]. This complication results in venous ischemia and gangrene of the affected limb presenting as massive edema, exquisite pain, and violaceous discoloration with visible skin blebs and bullae, in some cases progressing to compartment syndrome with a high associated rate of amputation and death [5,11]. May-Thurner syndrome has always been a diagnostic challenge as there is no single imaging criterion for the diagnosis [11]. It has been considered a diagnosis of exclusion where other differentials like recent catheterization, radiation, surgery, direct trauma, and malignancy have been ruled out [3].

Our patient had presented at Stage III of the condition where she had developed frank iliofemoral thrombosis. Her symptomatology was not magnified due to the fact that she had established extensive collateral circulation indicating that the condition may have been more chronic. The acute presentation may have been precipitated by her recent psychiatric decompensation and infection that led to immobility, dehydration, and overall hypercoagulability with the hyper-viscose state.

Noninvasive diagnostic modalities, including venous duplex ultrasound that has high sensitivity and specificity, can be used as initial screening tools in the absence of thrombus [12]. Noninvasive imaging that can be utilized in addition to duplex ultrasound includes a CT venogram and an MR venogram [6,8,12]. For patients with extensive ilio-caval deep vein thrombosis, removal of the thrombus is necessary to uncover the stenotic venous lesion to make the appropriate diagnosis [4,12]. Imaging findings consistent with May-Thurner's diagnosis include more than 50% luminal stenosis visualized at the appropriate anatomic location as was seen in our patient after removal of clot burden [7,11]. Invasive venous imaging is considered both therapeutic and diagnostic. Catheter-based venography is warranted and is considered the gold standard for diagnosis when coupled with transvenous pressure measurements [6,7,11].

Over the past decade, intravascular ultrasound has been used as the standard for establishing the diagnosis and is considered a valuable tool during treatment. The use of a 20-MHz ultrasound transducer can accurately determine the size and morphology of the left common iliac vein along with verifying the presence of May-Thurner anatomy. This is achieved by the visualization of the more than 50% occluded lumen proximal to the intravascular ultrasound catheter [11]. In patients with iliofemoral deep vein thrombosis with near-total occlusion, early initiation of anticoagulation coupled with endovascular treatment is still considered the preferable mode of management when compared to anticoagulation alone [5,6,11]. Treatment options that focus on the reduction of clot burden by endovascular thrombolysis coupled with mechanical thrombectomy have shown a significant improvement in symptoms with reduction of complications [3,11]. The current standard of care continues to be initial clot lysis followed by continuous infusion of the thrombolytic for 24-48 h followed by intravascular stent placement in the iliac vein to prevent restenosis and compression [3,6,11]. At least three to six months of anticoagulation therapy are warranted in these patients [6,7].

## Conclusions

May-Thurner syndrome should be considered as a differential in patients who present with isolated left leg deep vein thrombosis with significant swelling and problems with ambulation. This, if not recognized and treated, can have significant life- and limb-altering complications including but not limited to the post-thrombotic syndrome, venous ischemia, compartment syndrome, and phlegmasia cerulea dolens. Early recognition coupled with an early endovascular intervention can significantly improve the quality of life and reduce morbidity as well as mortality in these patients.
